# Anterior cruciate ligament ganglion: case report

**DOI:** 10.1590/S1516-31802002000600009

**Published:** 2002-11-01

**Authors:** André Pedrinelli, Fábio Bonini Castellana, Ricardo Bragança de Vasconcellos Fontes, Rafael Ferreira Coelho, Luiz Álvaro de Menezes F°.

**Keywords:** Knee, Ganglion, Anterior cruciate ligament, Joelho, Ganglion, Ligamento cruzado anterior

## Abstract

**CONTEXT:**

A ganglion is a cystic formation close to joints or tendinous sheaths, frequently found in the wrist, foot or knee. Intra-articular ganglia of the knee are rare, and most of them are located in the anterior cruciate ligament. The clinical picture for these ganglia comprises pain and movement restrictions in the knee, causing significant impairment to the patient. Symptoms are non-specific, and anterior cruciate ligament ganglia are usually diagnosed through magnetic resonance imaging or arthroscopy. Not all ganglia diagnosed through magnetic resonance imaging need to undergo surgical treatment: only those that cause clinical signs and symptoms do. Surgical results are considered good or excellent in the vast majority of cases.

**CASE REPORT:**

A 29-year-old male presented with pain in the left knee during a marathon race. Physical examination revealed limitation in the maximum range of knee extension and pain in the posterior aspect of the left knee. Radiographs of the left knee were normal, but magnetic resonance imaging revealed a multi-lobed cystic structure adjacent to the anterior cruciate ligament, which resembled a ganglion cyst. The mass was removed through arthroscopy, and pathological examination revealed a synovial cyst. Patient recovery was excellent, and he resumed his usual training routine five months later.

## RESUMO

**CONTEXTO:**

Um ganglion é uma formação cística próxima a articulações ou bainhas tendinosas, freqüentemente encontrado no punho, pé e joelho. Ganglia intra-articular do joelho são raros, e a maioria destes está localizada no ligamento cruzado anterior. O quadro clínico destes ganglia é composto por dor e limitação de movimento do joelho, causando importante prejuízo ao paciente. Os sintomas são inespecíficos, e ganglia são geralmen-te diagnosticados por ressonância magnética ou artroscopia. Nem todos os ganglia diagnosticados por ressonância magnética precisam ser submetidos a cirurgia, mas apenas aqueles com sinais e sintomas clínicos. Os resultados cirúr-gicos são considerados bons ou excelentes na grande maioria dos casos.

**RELATO DE CASO:**

Um homem de 29 anos apresentou dor no joelho esquerdo durante uma corrida de maratona. O exame físico re-velou limitação da extensão máxima do joe-lho e dor na face posterior do joelho esquerdo. Radiografias do joelho esquerdo foram normais, mas a ressonância magnética revelou uma estrutura cística multilobulada adjacente ao ligamento cruzado anterior, que lembrava um ganglion. A massa foi removida por artroscopia, e o exame patológico revelou um cisto sinovial. A recuperação do paciente foi excelente, e ele retomou sua rotina de treina-mento normal cinco meses depois.

## INTRODUCTION

A ganglion is defined as a cystic formation, close to joints or tendinous sheaths, frequently found in the wrist, foot or knee. It can be single or multi-lobed, with clear gelatinous, colloid or mucinous content.^1^ Ganglia located at the dorsum of the foot or the wrist, and those close to the interphalangeal articulations are of easy clinical diagnosis, commonly found in clinical practice and usually called ‘synovial cysts’.^1^ However, deeper ganglia, such as those found in the forearm, in the periacetabular region or in the suprascapular notch and intra-articular knee ganglia, are difficult to diagnose clinically, especially when not palpable. These are accidentally found when magnetic resonance imaging scans^2,3^ or arthroscopy^4-7^ are performed.

Intra-articular ganglia of the knee are rare and most of them (62.6%) are located in the anterior cruciate ligament.^1^ Its etiology remains unknown. The clinical picture of knee ganglia consists of pain and sometimes restriction in the final degrees of extension.^4-7^ These symptoms are especially disturbing to the patient, which makes diagnosis and specific treatment necessary.

We report a case of anterior cruciate ligament ganglion in a 29-year-old patient.

## CASE REPORT

A 29-year-old male patient had been doing competition-level swimming from 1978 to 1998 and he started triathlon training in 1999. In the second half of 2000, while in a 1000-meter sprint at the end of a marathon race, he experienced pain in the posterior aspect of the left knee.

**Figure 1:**
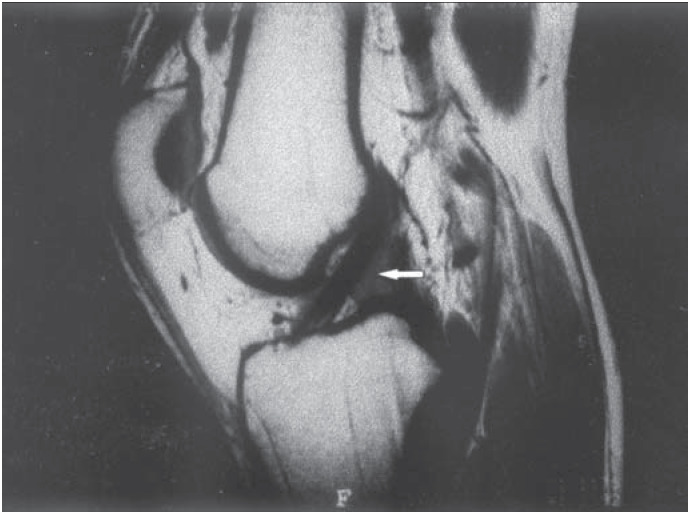
Magnetic resonance imaging of the patient's left knee. White arrow shows the anterior cruciate ligament ganglion.

**Figure 2:**
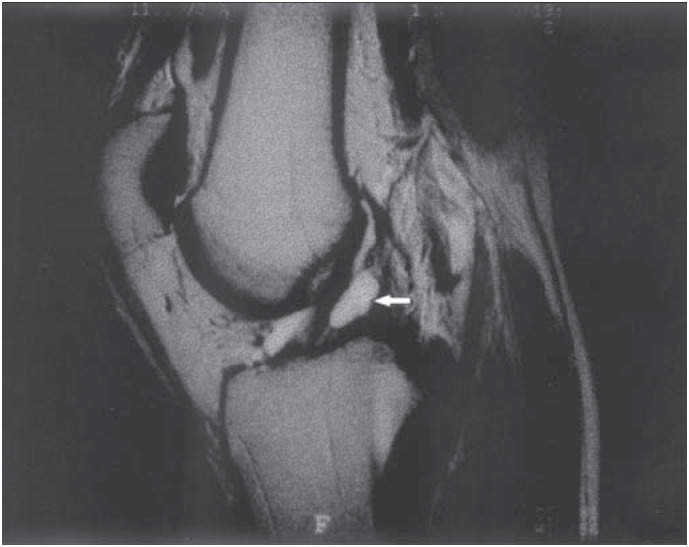
Magnetic resonance imaging of the patient's left knee. White arrow points to the anterior cruciate ligament ganglion.

The patient sought medical help in December 2000, and his physical examination revealed pain in the posterior aspect of the left knee and over the popliteal muscle, which worsened when this area was compressed, and a slight increase in volume of this region. He also presented limitation in the range of knee extension. Lachman, jerk, anterior drawer, valgus and varus stress tests were all negative, as were tests for meniscal lesions as well. Radiographs obtained in the anteroposterior and 30-degree lateral projections did not reveal any abnormalities. Magnetic resonance imaging revealed a multi-lobed cystic structure adjacent to the anterior cruciate ligament, measuring 3 × 0.5 centimeters, which resembled a ganglion cyst (*Figures 1 and 2*).

**Figure 3:**
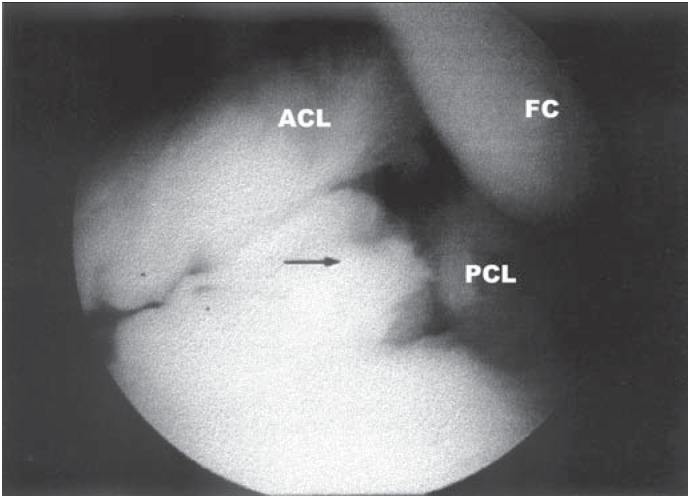
Arthroscopy view of the anterior cruciate ligament ganglion. Black arrow indicates the ganglion. FC: Femoral condyle. ACL: Anterior cruciate ligament. PCL: Posterior cruciate ligament.

The patient underwent knee arthroscopy in November 2000 and had the cystic structure removed (Figure 3). Pathological examination revealed that the mass was a synovial cyst, with a myxoid degeneration focus and light reactive inflammation.

The patient had an excellent recovery, without any pain on the second postoperative day. He also underwent physiotherapy, with emphasis on functional and motor recovery, and in December 2000 received permission to return to light jogging activities. After four months, the patient returned once more to the clinic, having already resumed his usual training routine, including triathlon competitions, without any symptoms.

## DISCUSSION

The anterior cruciate ligament ganglion is an uncommon clinical entity. Goldstein and Manacés^1^ reported that 42.1% of their patients related the beginning of their symptoms to some kind of traumatic event, and that 56.6% did not possess any trauma history. In the specific case of this patient, the repeated 1,000-meter sprints could be considered traumatic events. Authors who defend the traumatic hypothesis have explained that fusiform dilatation of the ligament fibers occurs secondary to hyaluronic acid infiltration, produced in response to trauma. On the other hand, a congenital anomaly may be the etiology behind ganglia in patients without traumatic history.^2,8^

The most frequently presented symptoms are pain and limitation in the range of knee extension. The data in the work of Goldstein and Manacés^1^ reveal that 79.5% of their patients with intra-articular knee ganglia complained of pain and 22.9% of limitation in the range of extension. However, these symptoms are non-specific, and can be present in several other intra-articular diseases, frequently making clinical diagnosis a difficult task. In consequence, the ganglion is usually encountered as a chance finding from magnetic resonance imaging or arthroscopy procedures. The same paper by Goldstein and Manacés states that 91.6% of the ganglia were chance findings and only 2.4% were palpable and thus clinically diagnosed.^1^ Magnetic resonance imaging is the safest method for diagnosing the anterior cruciate ligament ganglion,^2^ providing the exact location and dimensions of these ganglia, which vary from 5 to 40 mm, in a non-invasive manner. Furthermore, not all ganglia diagnosed through magnetic resonance imaging need to undergo surgical treatment: only those that cause clinical signs and symptoms do. Bui-Mansfield and Youngberg^2^ reported that 22% of their patients with in- tra-articular knee ganglia diagnosed through magnetic resonance imaging underwent arthroscopy later on. On the other hand, a ganglion encountered by chance during arthroscopy can be removed^1^ while carefully avoiding anterior cruciate ligament lesion. There are no reports of recurrence of intraarticular knee ganglia after surgical treatment, and the results are considered "good" or "excellent" in 94.7% of these cases.^1^
