# ERRATUM

**DOI:** 10.1590/S1516-31802003000200002

**Published:** 2003-03-05

In the article "**Anterior cruciate ligament gaglion: case report**", published in the edition dated 1 November 2002, volume 120, number 6, pages 195-197, the correct name of one of the authors is Luiz Álvaro de Menezes Filho and not Luiz Álvaro de Menezes Silva.

